# Utilization Pattern and Related Knowledge of Nasal Decongestants Among the General Population in Al-Qunfudah Governorate, Saudi Arabia: A Community-Based Cross-Sectional Study

**DOI:** 10.7759/cureus.53006

**Published:** 2024-01-26

**Authors:** Safa H Alkalash, Abdulaziz M Alsokani, Ahmed A Alrezqi, Abdullah A Alrashdi, Hassan A Alzubaidi, Alhassan H Alfaqeh, Mohammed A Alfaqih, Ahmed A Alhayli, Nawaf M Alsuhaymi, Mohammed Alessa, Khalid A Alfaqih

**Affiliations:** 1 Community Medicine and Health Care, Umm Al-Qura University, Al-Qunfudah, SAU; 2 Family Medicine, Menoufia University, Shebin Elkom, EGY; 3 Medicine and Surgery, Umm Al-Qura University, Al-Qunfudah, SAU

**Keywords:** utilization, patterns, side-effects, local allergic rhinitis, nasal obstruction treatment, nasal decongestant

## Abstract

Background: Nasal decongestants, like phenylephrine and pseudoephedrine, are commonly used to relieve nasal obstruction in conditions such as allergic rhinitis. They induce nasal passage dilation through vasoconstriction but can lead to serious side effects like hypertension and rebound congestion. Despite being easily accessible over the counter, their usage patterns and awareness of side effects are not well studied.

Objectives: The study aimed to assess the utilization pattern and public knowledge of nasal decongestants in Al-Qunfudah governorate, Saudi Arabia, in 2023.

Methods: This observational cross-sectional study assessed the utilization pattern of nasal decongestants among those who were 10 years of age and older and resided in Al-Qunfudah governorate and its villages. Data were collected in three months, from June to August 2023, using a self-administered survey that was disseminated among the general population at Al-Qunfudah governorate on different electronic platforms like Twitter (X Corp., San Francisco, CA, United States) and Snapchat (Snap Inc., Santa Monica, CA, United States). RStudio (version 4.3.0) was used for the statistical analysis. The knowledge score showed a non-normal distribution (Shapiro-Wilk test p value < 0.001). For normally distributed qualitative variables, the factors related to nasal decongestant use were assessed using Pearson's Chi-squared test. Fisher's exact test was applied when more than 20% of cells had frequencies less than 5. A generalized linear regression model was used to assess the independent predictors of higher knowledge scores. A p-value < 0.05 indicated statistical significance.

Results: Based on 410 responses, nearly 77% (n = 314) of the participants have ever used nasal decongestants. A total of 118 out of 314 (37.6%) used these medications twice daily for less than five days (81.2%, n = 255). A total of 192 (61.1%) participants used nasal decongestants based on physicians' prescriptions. Few respondents (12.9%, n = 53) and (33.2%, n = 136) correctly identified nasal mucosal ulceration and nasal dryness as adverse effects of prolonged nasal decongestants' use. However, 84.6% (n = 347) ignored their contraindications, and 55.1% (n = 226) had no idea about rebound congestion. Overall, participants displayed a moderate level of knowledge regarding nasal decongestants, with a median knowledge score of 5.0. Being a student (beta = 1.12, 95%CI, 0.19 to 2.05, p = 0.019) and being a female were independently associated with better knowledge scores (beta = 0.97, 95%CI, 0.40 to 1.54, p < 0.001). Those who ever used nasal decongestants (beta = 0.71, 95% CI: 0.07 to 1.34, p = 0.030) and those who used them three times a day (beta = 1.05, 95% CI: 0.11 to 1.99, p = 0.029) had higher knowledge scores.

Conclusion: More than two-thirds (76.6%) of the Al-Qunfudah general population in Saudi Arabia utilized nasal decongestants. The utilization pattern of nasal decongestants highlighted short-term usage for nasal obstruction. Despite the moderate level of knowledge of the general population about nasal decongestants, many gaps were noted regarding their systemic contraindications, side effects, and the risks of rebound congestion. A focus group discussion is advised to get a full and deep perception of the public regarding this common type of medication. Health education programs are recommended regarding this category of medications, warning them about ineffective self-medication.

## Introduction

Nasal decongestants are a group of medications commonly used in otorhinolaryngology and general medical practices to reopen the nasal airway and relieve the nasal obstruction that is seen in diseases such as allergic rhinitis, sinusitis, and upper respiratory tract infections [1]. They are available in both systemic and topical form, and they are sympathomimetics that exert their effect by working on adrenergic receptors. Examples include phenylephrine, pseudoephedrine, oxymetazoline, and xylometazoline [1]. This category of medications may cause congestion of the nose; shortly after use, a phenomenon called rebound congestion, or rhinitis medicamentosa, is caused by the stimulation of the beta-receptors. Also, the frequent use of topical decongestants may cause the patient to develop a tolerance and decrease the efficacy of the drug; this may be attributed to the downregulation of receptors. Both rebound congestion and tolerance will lead the patient to more frequent usage of the drug and eventually more side effects, causing rhinitis medicamentosa [2,3]. Hypothesizes that it’s caused by ischemia, other nasal mucosa, or decreased activity of the sympathetic system [3].

Patients' understanding of their prescribed medications regarding their names, purpose, dosage schedule, potential side effects, and any special instructions associated with the medication is essential for lowering prescription mistakes, enhancing adherence, and raising patient satisfaction [4]. Research has demonstrated that insufficient health literacy leads to poor health outcomes, especially when it comes to misinterpreting prescription usage instructions [5,6].

Nasal decongestants are commonly used and are easily accessible and available over the counter, but as before mentioned, they have many side effects [7]. A study from a large Saudi city, Jeddah, revealed that a significant percentage (over 50%) of adults used over-the-counter (OTC) medications in an improper and dangerous manner [8]. Additionally, few studies in Saudi Arabia detected inadequate knowledge about medication uses [9,10]. Thus, in this study, we aimed to measure the utilization pattern of nasal decongestants and their related knowledge among the general population of Al-Qunfudah governorate of Saudi Arabia due to the lack of studies done to cover such an important subject in this remote area.

## Materials and methods

Study design

An observational community-based cross-sectional study was conducted among the general population at Al-Qunfudah governorate, Saudi Arabia, over a period of three months, from June to August 2023.

Study population

The study participants were people aged 10 years or older who resided in the Al-Qunfudah governorate, Saudi Arabia.

Sample size

Epi-Info (Centers for Disease Control and Prevention, Atlanta, GA, USA) was used to estimate the minimum sample size required for data collection. Based on the total population of Al-Qunfudah governorate (300,516) [11] and the frequency of nasal decongestants' utilization in Saudi Arabia (45.1%) [8], at CI (95%) and margin of error (5%), the minimum sample size was 380.

Development and validation of the study survey

The study survey was designed in an electronic, self-administered format after scanning preexisting studies from a literature review [8,12], then reviewed and validated by family medicine and pharmacology consultants. It was elaborated in Arabic, which is the native language of the Saudi population, and comprised 33 questions organized in four sections. The first section included questions to assess the socio-demographic data, like age, gender, nationality, and marital status. The second section involved items about the utilization pattern of nasal decongestants as regards duration, frequency, and indications to use (nasal obstruction, common cold, itching, sneezing), as well as who prescribed them for the participants, whether a physician or pharmacist or by themselves. The third portion contains questions to assess participants' knowledge regarding the names of nasal decongestants in Arabic and their types, side effects, contraindications, and therapeutic benefits. A cover page was provided that illustrates both the purpose of the study and the consent of the participants.

The knowledge domain was based on 11 items with 17 correct answers. For each participant, a knowledge score was created by summing up the correct values of knowledge items (each correct item was assigned 1 and each incorrect item was assigned 0). Therefore, the total knowledge score ranged between 0 and 17, where higher scores indicated higher levels of knowledge.

A pilot study was conducted in May 2023 to verify the reliability of the questionnaire, and Google Forms (Google LLC, Mountain View, CA, USA) was used to develop the survey. The survey link was shared on Al-Qunfudah Snapchat (Snap Inc., Santa Monica, CA, USA) for 10 days as a pilot study involving 39 participants, which was done to ensure clear language and accurate data collection. In this pilot study, the response rate was estimated to be 93%. To assess the reliability of the questionnaire, the Cronbach's test was used. On a scale of 0 to 1, the acceptable range value for Cronbach's alpha is between 0.60 and 0.80 [13]. Cronbach’s alpha coefficient for the entire questionnaire was 0.71, which indicates its reliability. All items of the questionnaire were answered with no need for any modifications. The responses that were collected in the pilot study were excluded from the main data analysis.

Procedures for data collection

The main study data were collected from a convenience sample of 410 participants. An electronically self-administered questionnaire was used, as in the pilot study, and broadcasting of the survey link through Twitter (X Corp., San Francisco, CA, USA) and Snapchat, to the general population.

Ethical consideration

The ethical approval for this research was obtained from the scientific research ethics committee at Umm Al-Qura University (No. HAPO-02-K-012-2023-05-1623). It was ensured that there was no sharing of personal data. All the information was carefully labeled and handled to guarantee its privacy.

Statistical analysis

Rstudio (version 4.3.0) was used for the statistical analysis. Normality testing was applied to the knowledge score, which revealed a non-normally distributed variable (Shapiro-Wilk test p value < 0.001). Therefore, the knowledge score was presented as the median and interquartile range (IQR), and categorical variables were presented with an absolute and relative frequency distribution. Factors associated with nasal decongestants' utilization were evaluated using a Pearson's Chi-squared test when the qualitative variables were normally distributed. Fisher's exact test was used when more than 20% of cells had expected frequencies less than 5. The differences between sociodemographic groups in terms of the knowledge score were assessed using a Wilcoxon rank sum test for variables with two groups or a Kruskal-Wallis rank sum test for variables with three or more groups. Variables showing significant differences in the knowledge score were further used as independent variables in a generalized linear regression model to assess the independent predictors of higher knowledge scores. The Durbin-Watson statistic was used to test autocorrelation in the residuals from a statistical regression analysis. Results were presented as beta coefficients and 95% confidence intervals (95%CIs). A p value of < 0.05 refers to statistical significance.

## Results

Sociodemographic characteristics

Initially, we received 437 responses on the online platform. However, we excluded 13 responses from those who disagreed with participating and 14 responses from those residing outside Al-Qunfudah governorate. Therefore, we analyzed a total of 410 responses. The most prominent category in terms of region was Al-Qunfudah city and its surrounding villages (36.3%, n = 149). In the age distribution, the highest frequency was observed in the 41- to 60-year-old group (44.6%, n = 183). Among the participants, 52.7% (n = 216) were male. An overwhelming majority were Saudi nationals (99.0%, n = 406). The marital status with the highest representation was "married" individuals (67.6%, n = 277). In the education category, "university or above" stood out with 68.3% (n = 280). In terms of monthly income, "5,000 to 15,000 SAR" was the most common bracket (49.5%, n = 203). Among the participants, 87.8% (n = 360) were non-smokers, and within the subset of active smokers (6.8%, n = 28), 50.0% (n = 14) smoke "less than one packet" per day. When it comes to employment, the "government sector" had the highest frequency at 49.5% (n = 203) (Table 1).

**Table 1:** Sociodemographic characteristics of the study subjects SAR= Saudi Arabian Riyal.

Characteristics	N= 410	(%)
Residence		
Al-Qunfudah and its surrounding villages	149	(36.3%)
Al-Mudhaylif and its surrounding villages	53	(12.9%)
Al-Quoz and its surrounding villages	36	(8.8%)
Hali and its surrounding villages	7	(1.7%)
Al-Ardiyat	141	(34.4%)
Sabia / Bani Essa	24	(5.9%)
Residence area		
Rural	261	(63.7%)
Urban	149	(36.3%)
Age (year)		
10-18	18	(4.4%)
19-30	96	(23.4%)
31-40	105	(25.6%)
41-60	183	(44.6%)
More than 60	8	(2.0%)
Gender		
Male	216	(52.7%)
Female	194	(47.3%)
Nationality		
Saudi	406	(99.0%)
Non-Saudi	4	(1.0%)
Marital Status		
Single	111	(27.1%)
Married	277	(67.6%)
Divorced	16	(3.9%)
Widow	6	(1.5%)
Educational Level		
Below secondary	28	(6.8%)
Secondary	102	(24.9%)
University or above	280	(68.3%)
Monthly Income (SAR)		
Less than 5000	132	(32.2%)
5000 to 15000	203	(49.5%)
More than 15000	75	(18.3%)
Employment Status		
Non-worker	63	(15.4%)
Student	70	(17.1%)
Government sector	203	(49.5%)
Private sector	17	(4.1%)
Military sector	23	(5.6%)
Retired	34	(8.3%)
Smoking Status		
Non-smoker	360	(87.8%)
Current smoker	28	(6.8%)
Ex-smoker	22	(5.4%)
For active smokers, the number of packets smoked per day (n=28)		
Less than one	14	(50.0%)
One to two	13	(46.4%)
More than two	1	(3.6%)

Frequency of nasal decongestant use and associated factors

In general, 314 participants indicated using nasal decongestants, which represented 67.6% of the sample (95% CI, 72.1 to 80.5). Nasal decongestant utilization was found to be significantly higher in Hali and its surrounding villages (100.0%, n = 7) and Al-Ardiyat (85.1%, n = 120) compared to other regions (p = 0.008). The remaining sociodemographic variables were not significantly associated with nasal decongestants' usage (Table 2).

**Table 2:** Factors associated with the utilization of nasal decongestants A p-value less than 0.05 explained the presence of a statistically significant difference; # Chi squared test; * Fisher's exact test.

Characteristics	Nasal Decongestants Utilization											p-value
	No							Yes				
	N=96				(%)			N=314			(%)	
Residence												
Al-Qunfudah and its surrounding villages	38				(25.5%)			111			(74.5%)	0.008^*^
Al-Mudhaylif and its surrounding villages	15				(28.3%)			38			(71.7%)	
Al-Quoz and its surrounding villages	14				(38.9%)			22			(61.1%)	
Hali and its surrounding villages	0				(0.0%)			7			(100.0%)	
Al-Ardiyat	21				(14.9%)			120			(85.1%)	
Sabia / Bani Essa	8				(33.3%)			16			(66.7%)	
Area of residence												
Rural	58				(22.2%)			203			(77.8%)	0.450^#^
Urban	38				(25.5%)			111			(74.5%)	
Age (year)												
10-18	6				(33.3%)			12			(66.7%)	0.099^*^
19-30	30				(31.3%)			66			(68.8%)	
31-40	20				(19.0%)			85			(81.0%)	
41-60	37				(20.2%)			146			(79.8%)	
More than 60	3				(37.5%)			5			(62.5%)	
Gender												
Male	56		(25.9%)					160			(74.1%)	0.205^#^
Female	40		(20.6%)					154			(79.4%)	
Nationality												
Saudi	95					(23.4%)		311			(76.6%)	0.999^*^
Non-Saudi	1					(25.0%)		3			(75.0%)	
Marital Status												
Single	32					(28.8%)		79	(71.2%)			0.290^*^
Married	58					(20.9%)		219	(79.1%)			
Divorced	4					(25.0%)		12	(75.0%)			
Widow	2					(33.3%)		4	(66.7%)			
Educational Level												
Below secondary	8						(28.6%)	20		(71.4%)		
Secondary	27						(26.5%)	75		(73.5%)		0.506^#^
University or above	61						(21.8%)	219		(78.2%)		
Monthly Income (SAR)												
Less than 5000	39				(29.5%)			93	(70.5%)			0.073^#^
5000 to 15000	45				(22.2%)			158	(77.8%)			
More than 15000	12				(16.0%)			63	(84.0%)			
Smoking Status												
Non-smoker	85	(23.6%)						275	(76.4%)			0.450^*^
Current smoker	8	(28.6%)						20	(71.4%)			
Ex-smoker	3	(13.6%)						19	(86.4%)			
Employment Status												
Non-worker	9			(14.3%)				54	(85.7%)			0.050^#^
Student	21	(30.0%)						49	(70.0%)			
Government sector	43	(21.2%)						160	(78.8%)			
Private sector	8	(47.1%)						9	(52.9%)			
Military sector	5	(21.7%)						18	(78.3%)			
Retired	10	(29.4%)						24	(70.6%)			

**Figure 1:**
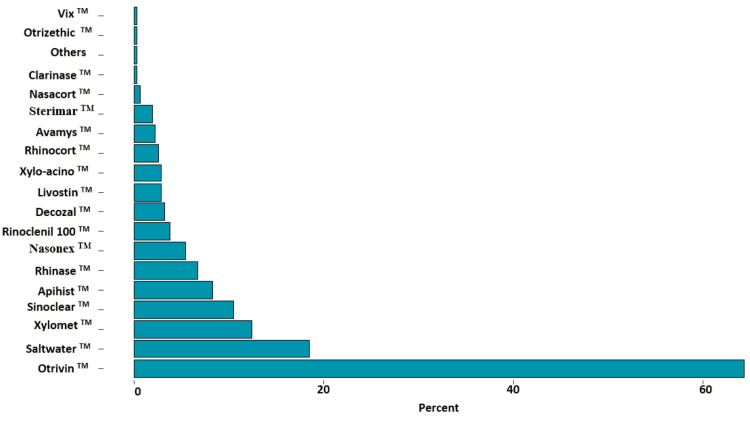
Percentages of types of the used nasal decongestants among their users (N=314)

The most common types of nasal decongestants used by the respondents included Otrivin^TM^ (xylometazoline hydrochloride and ipratropium bromide) (64.3%, n = 202) and saltwater (18.5%, n = 58) (Figure 1).

The utilization pattern of nasal decongestants

Focusing on the users of nasal decongestants (76.6%, n = 314), many respondents used nasal decongestants for less than five days (81.2%, n = 255). Additionally, the majority of the users reported using the medication twice daily (37.6%, n = 111). The primary reason for using nasal decongestants was "nasal obstruction" (65.9%, n = 207). A total of 151 respondents (representing 48.1% of nasal decongestants' users) indicated the use of other medications alongside nasal decongestants, of whom the most common supplementary medication was "oral antihistamine" (49.7%, n = 75). A notable portion of participants reported receiving prescriptions for nasal decongestants from a "physician" (61.1%, n = 192). Most nasal decongestant users reported receiving advice on how to use nasal decongestants (82.5%, n = 259). More than half of the study respondents purchased nasal decongestants from a "pharmacy" (58.6%, n = 184), making it the primary site for acquisition. Most participants (93.6%, n = 294) reported using "one bottle per month." Additionally, a substantial proportion (93.6%, n = 294) noted that their symptoms improved with nasal decongestant usage. Only 15.9% of the participants (n = 50) reported having received a medical diagnosis for nasal decongestant-related problems (Table 3).

**Table 3:** The utilization pattern of nasal decongestants among their users

Characteristics	N= 314	(%)
Duration of using nasal decongestants		
Less than 5 days	255	(81.2%)
5-15 days	28	(8.9%)
16-30 days	8	(2.5%)
2-6 months	7	(2.2%)
7-12 months	16	(5.1%)
Frequency of using nasal decongestants per day		
1 time/day	83	(26.4%)
2 times/day	118	(37.6%)
3 times/day	38	(12.1%)
4 times/day	5	(1.6%)
Only with symptoms	70	(22.3%)
Causes of using nasal decongestants		
Sneezing	22	(7.0%)
Common cold	126	(40.1%)
Nasal obstruction	207	(65.9%)
Itching	14	(4.5%)
Rhinosinusitis	79	(25.2%)
Allergic Rhinitis	27	(8.6%)
Others	5	(1.6%)
Hay fever	3	(1.0%)
Persons who recommended nasal decongestant for you		
Physician	192	(61.1%)
Pharmacist	56	(17.8%)
Family	22	(7.0%)
Friends	11	(3.5%)
My self	28	(8.9%)
Internet	5	(1.6%)
Received any advice on how to use nasal decongestants		
No	55	(17.5%)
Yes	259	(82.5%)
The main site for purchasing nasal decongestants		
Pharmacy	184	(58.6%)
Primary healthcare center	31	(9.9%)
Hospital	99	(31.5%)
Number of bottles used per month		
One	294	(93.6%)
Two	17	(5.4%)
Three	1	(0.3%)
Four or more	2	(0.6%)
Symptoms improved with the use of nasal decongestants		
No	20	(6.4%)
Yes	294	(93.6%)
Ever diagnosed by a physician for any complications due to nasal decongestants		
No	264	(84.1%)
Yes	50	(15.9%)
Other medications used with nasal decongestants (N=151)		
Paracetamol	46	(30.5%)
Oral antihistamine	75	(49.7%)
Others	48	(31.8%)

Participants responses to knowledge items about nasal decongestants

More than two-thirds (75.9%, n = 311) acknowledged that nasal decongestants relieve the symptoms, but only 20.5% (n = 84) mentioned that these medications cause rebound congestion when used for more than five days. A significant portion of the population recognized that they are used for symptomatic treatment only, but the population did not know whether they are safe to use in children (57.3%, n = 235) or not. Additionally, most (67.8%, n = 278) did not know about the absorption of nasal decongestants by the systemic circulation. Participants also correctly identified that patients should not share a bottle of nasal decongestants with others due to the risk of infection (56.8%, n = 233), while only 13.9% (n = 57) indicated that nasal decongestants are not among the major classes of drugs responsible for the poisoning and death of children under five years of age. Furthermore, a small proportion correctly identified the side effects of prolonged use of topical nasal decongestants, including nasal dryness (33.2%, n = 136) and nasal mucosal ulcers (12.9%, n = 53). The majority of participants were unaware of the contraindications (84.6%), and only a few were familiar with some, with hypertension being the most recognized (12.9%) (Table 4).

**Table 4:** Participants responses to knowledge questions about nasal decongestants *An asterisk indicates a correct answer.

Knowledge items	N= 410	(%)
Nasal decongestants relieve the symptoms of congestion and rhinitis associated with the common cold		
No	23	(5.6%)
Yes*	311	(75.9%)
Do not know	76	(18.5%)
Nasal decongestants cause rebound congestion when used for more than five days		
No	100	(24.4%)
Yes*	84	(20.5%)
Do not know	226	(55.1%)
Nasal decongestants are used for symptomatic treatment only		
No	51	(12.4%)
Yes*	262	(63.9%)
Do not know	97	(23.7%)
Nasal decongestants safe to use in children		
No	56	(13.7%)
Yes*	119	(29.0%)
Do not know	235	(57.3%)
Topical agents are not effective because they are weakly absorbed by the systemic circulation		
No*	54	(13.2%)
Yes	78	(19.0%)
Do not know	278	(67.8%)
Patients can share a bottle of Nasal decongestants s with other people because there is no risk of infection		
No*	233	(56.8%)
Yes	55	(13.4%)
Do not know	122	(29.8%)
Nasal decongestants are responsible for poisoning and death of children under five years old		
No*	57	(13.9%)
Yes	47	(11.5%)
Do not know	306	(74.6%)
Side effects of prolonged use of topical nasal decongestants		
Nasal dryness*	136	(33.2%)
Ulceration of the nasal mucosa*	53	(12.9%)
Do not know	221	(53.9%)
The best position of using topical nasal decongestants		
To tilt one’s head forward, dripping drops in the amount indicated *	272	(66.3%)
Person can put the drops in any positions with no special positions.	37	(9.0%)
Do not know	101	(24.6%)
Nasal instillation of saline is an adjuvant for nasal decongestants and effective treatment		
No	17	(4.1%)
Yes*	222	(54.1%)
Do not know	171	(41.7%)
Contraindications of systemic form of nasal decongestants		
Do not know	347	(84.6%)
Hypertension*	53	(12.9%)
Hyperthyroidism*	20	(4.9%)
Seizure*	18	(4.4%)
Glaucoma*	15	(3.7%)
Difficulties in urination*	12	(2.9%)
Ischemic heart disease*	11	(2.7%)
Prostatic diseases*	9	(2.2%)

Characteristics of the knowledge score and the associated factors

**Figure 2:**
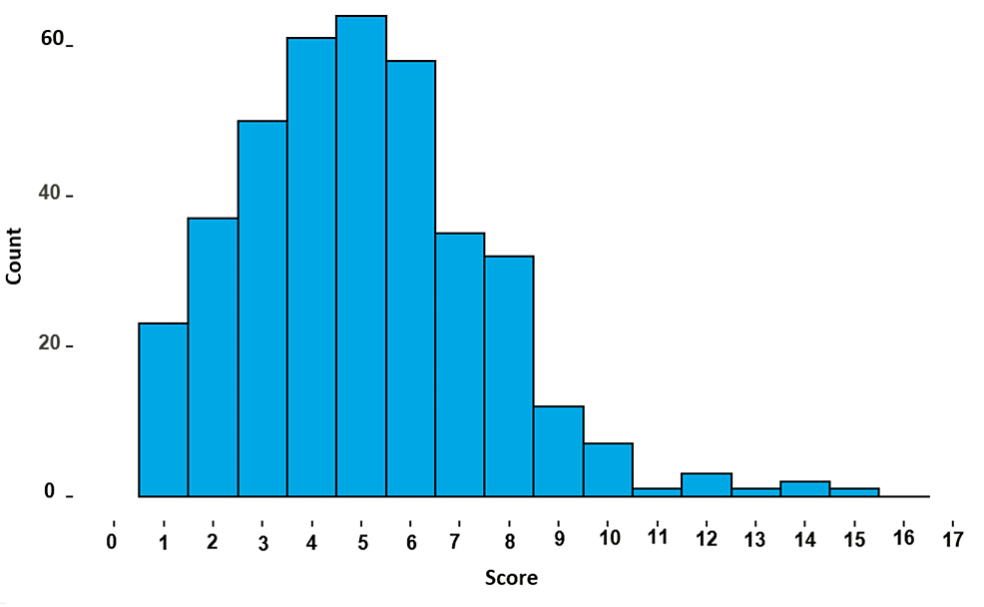
A histogram depicting the frequency distribution of the knowledge median score

The median knowledge score for all the participants was 5.0 (IQR = 3.0 to 6.0), with a minimum of 0 and a maximum of 17 (Figure 2).

Females had a significantly higher median value of the knowledge score (median = 5.0, IQR = 4.0 to 7.0) compared to males (median = 4.0, IQR = 2.0 to 6.0, p < 0.001). Furthermore, knowledge scores were significantly higher in those working in government sectors (p = 0.027), those who have ever used these medications (p <0.001), and those with a daily frequency of three times (p <0.001)(Table 5).

**Table 5:** Factors associated with higher knowledge scores regarding nasal decongestant utilization among the study participants A p-value less than 0.05 explained the presence of a statistically significant difference. IQR = Interquartile range. *The analysis was performed using a Wilcoxon rank sum test; otherwise, a Kruskal-Wallis rank sum test was used. NCDs = nasal decongestants. SAR= Saudi Arabian Riyal.

Characteristics	Median	(IQR)	p-value
Residence			
Al-Qunfudah and its surrounding villages	4.00	(3.00, 6.00)	0.192
Al-Mudhaylif and its surrounding villages	4.00	(3.00, 5.00)	
Al-Quoz and its surrounding villages	6.00	(3.00, 7.00)	
Hali and its surrounding villages	5.00	(4.50, 5.00)	
Al-Ardiyat	5.00	(3.00, 7.00)	
Sabia / Bani Essa	4.00	(2.00, 6.00)	
Area of residence*			
Rural	5.00	(3.00, 6.00)	0.226
Urban	4.00	(3.00, 6.00)	
Age (year)			
10-18	3.50	(1.00, 6.00)	0.691
19-30	5.00	(3.00, 7.00)	
31-40	5.00	(3.00, 6.00)	
41-60	5.00	(3.00, 6.00)	
More than 60	5.00	(3.75, 6.25)	
Gender*			
Male	4.00	(2.00, 6.00)	<0.001
Female	5.00	(4.00, 7.00)	
Nationality*			
Saudi	5.00	(3.00, 6.00)	0.258
Non-Saudi	7.00	(5.00, 8.00)	
Marital Status			
Single	4.00	(2.00, 6.50)	0.640
Married	5.00	(3.00, 6.00)	
Divorced	4.50	(3.75, 7.00)	
Widow	4.50	(3.25, 5.75)	
Educational Level			
Below secondary	5.00	(3.00, 6.00)	0.055
Secondary	4.00	(2.00, 6.00)	
University or above	5.00	(3.00, 7.00)	
Monthly Income (SAR)			
Less than 5000	5.00	(3.00, 6.25)	0.794
5000 to 15000	5.00	(3.00, 6.00)	
More than 15,000	5.00	(3.00, 7.00)	
Smoking Status			
Non-smoker	5.00	(3.00, 6.00)	0.481
Current smoker	4.50	(3.00, 6.00)	
Ex-smoker	4.00	(3.00, 5.00)	
Employment Status			
Non-worker	5.00	(3.00, 6.00)	0.027
Student	5.00	(3.00, 8.00)	
Government sector	5.00	(3.00, 6.00)	
Private sector	4.00	(2.00, 5.00)	
Military sector	3.00	(2.00, 6.00)	
Retired	4.00	(3.00, 5.00)	
Ever used nasal decongestants			
No	3.50	(1.00, 6.00)	<0.001
Yes	5.00	(3.00, 6.75)	
Duration of using NDCs			
Less than 5 days	5.00	(3.00, 6.00)	0.235
5-15 days	4.00	(3.00, 6.00)	
15-30 days	5.50	(4.75, 7.00)	
2-6 months	4.00	(2.50, 5.00)	
7-12 months	6.00	(5.50, 8.00)	
2-5 years	5.00	(5.00, 6.00)	
More than 5 years	3.00	(2.50, 5.00)	
Frequency of using NDCs per day			
1 time/day	5.00	(3.00, 6.25)	<0.001
2 times/day	5.00	(4.00, 6.75)	
3 times/day	6.00	(5.00, 7.00)	
4 times/day	5.00	(4.00, 5.00)	
Only with symptoms	4.00	(2.00, 6.00)	

On the multivariable analysis, a Durbin-Watson (D-W) test indicated that there was no correlation among residuals (D-W statistic = 2.144, p = 0.172). Additionally, there was no risk of multicollinearity since the variance inflation factor (VIF) values were below the threshold of 5 (VIF = 1.337 for gender, 1.403 for employment status, 1.230 for the use of nasal decongestants, and 1.321 for the frequency of nasal decongestants' usage). Being a student (beta = 1.12, 95% CI: 0.19 to 2.05, p = 0.019) and being a female were independently associated with better knowledge scores (beta = 0.97, 95% CI: 0.40 to 1.54, p < 0.001). Those who ever used nasal decongestants (beta = 0.71, 95% CI: 0.07 to 1.34, p = 0.030) and those who used them three times per day (beta = 1.05, 95% CI: 0.11 to 1.99, p = 0.029) were associated with good knowledge scores (Table 6).

**Table 6:** Predictors of higher knowledge scores regarding nasal decongestant utilization among the study participants A p-value less than 0.05 explained the presence of a statistically significant difference. CI = Confidence Interval. NCDs = nasal decongestants.

Characteristics	Beta	95% CI	p-value
Gender			
Male	Reference	Reference	
Female	0.97	0.40, 1.54	<0.001
Employment status			
Non-worker	Reference	Reference	
Student	1.12	0.19, 2.05	0.019
Government sector	0.39	-0.36, 1.13	0.311
Private sector	-0.53	-2.00, 0.93	0.476
Military sector	-0.25	-1.56, 1.07	0.711
Retired	-0.21	-1.35, 0.94	0.726
Ever used nasal decongestants			
No	Reference	Reference	
Yes	0.71	0.07, 1.34	0.030
Frequency of using NDCs per day			
1 time/day	Reference	Reference	
2 times/day	0.31	-0.35, 0.97	0.360
3 times/day	1.05	0.11, 1.99	0.029
4 times/day	1.11	-1.15, 3.37	0.338
Only with symptoms	-0.6	-1.27, 0.07	0.081

## Discussion

This research endeavor sought to examine the utilization pattern and knowledge pertaining to nasal decongestion medications among a cohort of 410 participants hailing from both rural and urban areas within Al-Qunfudah governorate, Saudi Arabia. The demographic characteristics of this study sample revealed a predominantly male, Saudi, and married population, aged between 41 and 60 years, possessing academic qualifications, gainfully employed, and residing more in Al-Qunfudah city and its surrounding suburban areas. These characteristics are due to the virtual mode of dissemination of the study instrument (Twitter and Snapchat).

This study revealed that 76.6% of the study subjects have ever utilized nasal decongestants. This percentage of utilization is like that reported by Alyahya et al.; they found that 68.5% of Saudi individuals had taken nasal decongestants anytime in their lives, 66.5% had done so with a prescription, and 31.5% had not [9]. The frequency of nasal decongestants' utilization is higher in comparison with that recorded by Alharthi and his associates in their study [8]. Their study, which targeted the Saudi general population in different Saudi regions, found that 45.1% use nasal decongestants. Further research is recommended, using in-person survey methods and a more representative sample from each area of interest (through cluster sampling).

The most popular kind of nasal decongestant documented by the study subjects was Otrivin^TM^, as it was utilized by 64.3% (n = 202). The key reasons for this finding may be due to the easy accessibility of this type of nasal decongestant as an over-the-counter medication, which is both cheap and more commonly prescribed by healthcare providers [7]. Despite there being no significant relation between those in rural or urban regions in terms of nasal decongestants' utilization (p = 0.450), participants from Hali and its surrounding villages (100.0%, n = 7) and Al-Ardiyat (85.1%, n = 120) recorded higher utilization percentages compared to other regions (p = 0.008). This statistical significance is attributed to the way the sample was calculated, as the sample calculation was based on the total population of Al-Qunfudah Governorate and not according to the different regions. The Hali region shows a disproportionate prevalence due to the small number of participants. Wojas et al. reported that nasal decongestants were used more among those living in urban areas [14]. This difference between both studies' findings may be due to differences in the studies' samples' inclusion criteria, as their study was done among patients suffering from allergic rhinitis in Poland.

Regarding the utilization pattern of nasal decongestants, most of the study sample (81.2%, n = 255) used them fewer than five days per week, and 37.6% (n = 118) utilized nasal decongestants twice daily. The most common indications for nasal decongestants' use were the presence of nasal obstruction (65.9%, n = 207) or common cold (40.1%, n = 126), and the majority by prescription (61.1%, n = 192). Many subjects (93.6%, n = 294) did not use more than one bottle per month. Finally, only 50 participants (15.9%) have ever developed complications and need medical management. This utilization pattern of nasal decongestants is like that recorded by Alharthi et al. and Alyahya et al. [8,9]. Also, a study in the United Arab Emirates revealed that 50% of the study sample utilized nasal decongestants due to the common cold [15]. On the other hand, an Indian study was done on patients who were utilizing topical nasal decongestants and were admitted to the Department of Ears, Nose, and Throat (ENT). The researchers detected that only 46.7% of people use nasal decongestants upon doctor prescription, and only 13.3% of them use the drops for fewer than 10 days, while 40% of the patients have been utilizing the drops for longer than three months [16]. In the north of Iran, a study by Parvinroo et al. revealed that 30.6% of the study cases self-medicated their rhinosinusitis, and the three most often used chemical medications were decongestants (10.3%), analgesics (55.2%), and antibiotics (75.9%) [17]. Another study done in Italy assessed community pharmacists' perceptions regarding misuse of nasal medications. It showed that up to 44.4% of cases had sympathomimetic amine dosages that were greater than advised, and up to 31.9% had usage periods longer than five days [18]. The main cause of the variance in the utilization pattern of nasal decongestants may be attributed to the cultural differences and different sample characteristics in the studies; however, this utilization pattern needs more attention.

On assessing knowledge regarding nasal decongestants' uses and adverse effects, the study subjects had adequate knowledge about some facts about nasal decongestants' use, while there was a gap in the others. More than two-thirds (75.9%, n = 311) knew about nasal decongestants’ indication, which is to relieve nasal obstruction in cases of common cold; it should be used for symptomatic relief only, as recorded by 262 subjects (63.9%); a total of 272 (66.3%) knew well about the correct position to administer nasal decongestants; and more than half of the sample could recognize that saline nasal drops are an effective alternative for nasal decongestants. These findings agreed with those obtained by Gill et al. [15] in their study, which reported that 50% of the study sample accurately knew that the common cold is the most common indication for using nasal decongestants.

The gap in knowledge about nasal decongestants among the studied population was more obvious as regards the occurrence of rebound congestion when used for more than five days, as 226 subjects (55.1%) did not know this information. Also, more than half (57%, n = 235) did not know about its safety to be used in children; 278 (67.8%) could not identify the effectiveness of topical nasal decongestants. A total of 221 (53.9%) denied the side effects of prolonged nasal decongestant use, and most participants (84.6%, n = 347) denied the contraindications for this category of medications. Similar to these findings, Al-Mutairi et al. [10] found that 83.4% of their study subjects were not aware of the side effects of nasal decongestants. Therefore, health education campaigns should be provided for the public, involving information about indications, contraindications, and side effects of nasal decongestants, focusing on correct medication use, and discouraging self-medication [19]. Furthermore, updating the knowledge of healthcare providers and encouraging them to counsel patients while prescribing medications to them.

Upon analysis of the collected data, it was observed that participants achieved a median knowledge score of 5.0 out of 17.0, indicative of a moderate level of understanding about nasal decongestants. In agreement with Rajasekaran and Ghosh [16], in their Indian study on nasal decongestant users, they detected a relatively low level of usage and knowledge about drugs. Despite the differences in both studies' settings and samples' characteristics, the issue of inadequate public knowledge regarding nasal decongestants and the poor utilization pattern is common. This makes us recommend that researchers from different areas worldwide establish different studies about the misuse of nasal decongestants and other over-the-counter medications that may have bad consequences for public health globally.

Females, students, those who ever used nasal decongestants, and those who used these medications three times per day could predict good knowledge regarding nasal decongestants (p-values are <0.001, 0.019, 0.030, and 0.029, respectively). This outcome is like the results extracted by Al-Mutairi et al., who concluded that female participants were significantly knowledgeable about the correct duration of nasal decongestant use [10]. The same study also indicated that the participants' knowledge about nasal decongestants was higher among those who had already used these medications than the others [10]. This outcome reflects that nasal decongestant users were keen to know about their medications in use.

Limitations and strengths

This study has a few drawbacks. Primarily, the study cohort was derived from a comparatively confined social demographic, potentially constraining the generalizability of our conclusions. Moreover, this survey was applied electronically, which exclusively limits its utilization to educated people. The convenience sample was associated with maldistribution of the target sample, but this could be overcome by using the cluster sampling approach. Despite the previously recorded restrictions, this study has its own strengths, as it may guide policymakers and healthcare providers to design health education messages and establish awareness campaigns based on the reported gaps in public knowledge about nasal decongestants. Furthermore, this study can guide other researchers to start a new series of investigations about different types of over-the-counter medications in this relatively isolated area that has limited healthcare facilities.

## Conclusions

In conclusion, the utilization percentage of nasal decongestants among the Al-Qunfudah general population in Saudi Arabia is 76.6%. Moderate knowledge of nasal decongestants was obvious among the general population of Al-Qunfudah; however, there were gaps in knowledge regarding their risk of rebound congestion and other potential side effects. Consequently, comprehensive public awareness programs are highly recommended, emphasizing the importance of procuring nasal decongestants through licensed medical practitioners and educating the public about the potential side effects and specific indications governing the judicious use of these pharmaceuticals. Further research is recommended using different study designs to get deeper information about public perceptions regarding the use of nasal decongestants, specifically among those who self-medicate with them. Research is also targeting health care providers, focusing on general practitioners, family, and otorhinolaryngology physicians to assess their preparedness to educate people about the rational use of over-the-counter medications, including nasal decongestants.
